# Alcohol use and sexual risk behaviors in the Armed Forces of the Democratic Republic of the Congo

**DOI:** 10.1186/s12889-019-7794-x

**Published:** 2019-10-28

**Authors:** Bonnie Robin Tran, Anthony Davis, Margo Sloan, Carol Macera, Anthony Mutombe Mbuyi, Gilbert Kurhgnga Kabanda

**Affiliations:** 1 Department of Defense HIV/AIDS Prevention Program, San Diego, California USA; 2 Leidos Inc, Reston, Virginia USA; 3 Armed Forces of the Democratic Republic of the Congo, Kinshasa, Democratic Republic of the Congo

**Keywords:** Alcohol, Sexual risk behaviors, HIV, Military populations

## Abstract

**Background:**

Alcohol misuse is an important contributor to sexual acquisition and transmission of HIV in military communities. This cross-sectional study quantified the prevalence of probable problematic alcohol use among male service members in the Armed Forces of the Democratic Republic of the Congo (FARDC), identified associated factors, and investigated associations of alcohol misuse with risky sexual behaviors.

**Methods:**

Participants included 2549 active duty male soldiers ≥ 18 years old. Data were collected via computer-assisted personal-interview from October 2013–April 2014. The Alcohol Use Disorders Identification Test (AUDIT) was used to identify probable problematic alcohol use (AUDIT score ≥ 8) compared to no/low-risk alcohol use (AUDIT score ≤ 7). Bivariate logistic regressions were used to identify factors associated with probable problematic alcohol use. Several multivariable logistic regressions (adjusted for age, marital status, education level) were used to examine associations of probable problematic alcohol use with risky sexual behaviors. Tests were two sided; statistical significance was defined as *p* < 0.05.

**Results:**

Fifteen percent of men screened positive for probable problematic alcohol use. The odds of probable problematic alcohol use were elevated among men who were single and living with a partner (OR = 1.66; 95% CI = 1.24–2.21), ranked as a non-commissioned officer [NCO] (OR = 1.40; 95% CI = 1.10–1.77), and in the 30–39 and 40–49 age groups (OR 30–39 age group = 2.17; 95% CI = 1.56–3.02; OR 40–49 age group = 1.79; 95% CI = 1.26–2.55). Probable problematic alcohol use was associated with increased odds of having sex with a sex worker (SW), having multiple sexual partners, and participating in transactional sex (aOR sex with a SW = 2.36; 95% CI = 1.78–3.13; aOR multiple sexual partners = 2.08; 95% CI = 1.66–2.60; aOR transactional sex = 1.99; 95% CI = 1.59–2.50).

**Conclusions:**

Results emphasize the need to address alcohol use in the FARDC and integrate alcohol abuse education into HIV prevention programs among male service members. Alcohol abuse prevention efforts should target men who are 30–49 years of age, unmarried, and ranked as a NCO. Messages and interventions to reduce alcohol misuse in relation to risky sexual behaviors are needed.

## Background

In 2017, UNAIDS reported that approximately 36.9 million [31.1–43.9 million] people worldwide were living with HIV. Additionally, 1.8 million [1.4–2.4 million] new infections occurred, and 940,000 [670,000-1,300,000] people died from AIDS-related causes [1]. These estimates have declined substantially from peak estimates of 3.4 million [2.6–4.4 million] for new HIV infections in 1996, and 1.9 million [1.4–2.7 million] for AIDS-related deaths in 2004. However, despite tremendous progress in HIV prevention, care, and treatment that have attributed to decreasing numbers, challenges in achieving epidemic control persist. In particular, the number of new HIV infections is not declining fast enough: prevention efforts to avert new HIV infections need to be strengthened to match the number of HIV positive people placed on treatment [1]. Furthermore, focusing on populations that may be more vulnerable to HIV infection and identifying factors that are associated with infection in these populations must remain a public health priority.

In particular, military populations may be at higher risk for HIV. Service members are predominantly young, vulnerable to peer pressure, highly mobile, and often sent away for lengthy deployments [2, 3]. To alleviate stress and loneliness during deployments, studies have shown military personnel to engage in risky sexual behaviors (such as having sex with sex workers and multiple partners) during local or foreign deployments [4]. Other research has shown HIV prevalence to be substantially higher in some military forces compared to the general population [5, 6]. Similar to the HIV policy in the US military [7], many militaries around the world also do not allow recruits who test HIV positive to enter service. Of concern is that military personnel are entering the forces HIV negative but are becoming HIV positive during their service. Characterizing key factors that place service members at higher risk for HIV infection during service is critical to develop an effective response to HIV.

Alcohol misuse has been identified as an important driver of the HIV epidemic in Sub-Saharan Africa. Meta analyses and systematic reviews have concluded that alcohol misuse is associated with an elevated risk of HIV infection by increasing risky sexual behaviors [8–13], such as sex with a sex worker, having multiple sexual partners, and participating in transactional sex. Engaging in sex with a female sex worker may increase the risk of HIV infection, as this group is disproportionally affected by HIV disease [14]. Multiple sexual partnerships may also elevate HIV risk as these relationships link individuals together and provide a pathway for the virus to move from one person to another. Additionally, the probability of exposure to primary HIV infection is amplified [15–17]. Transactional sex can also elevate HIV risk as individuals who engage in this behavior are more likely to experience different forms of abuse and violence [18, 19] or engage in other high risk sexual behaviors [20].

In military populations, several studies have shown rates of alcohol misuse to be higher among service members compared to the general population [21, 22]. Additionally, elevated odds of participating in risky sexual behaviors such as transactional sex, having multiple sexual partners, and having a higher number of sexual partners were observed among service members who exhibited patterns of excessive drinking [21, 23]. Alcohol misuse can lead to HIV infection through risky sexual behaviors, which ultimately impacts the health of service members, reduces operational readiness, and increases medical costs.

Several theories have been proposed to explain the associations between alcohol misuse and risky sexual behaviors. The alcohol myopia theory suggests that alcohol consumption can impair perception and thought, causing individuals to focus on cues that are most noticeable or important in the environment, while ignoring cues that are inhibitive or less salient as this would require more cognitive processing [24]. For example, in sexual situations, alcohol use may cause individuals to focus on immediate pleasure from the sexual act rather than the sexual partner’s disease risk. Conversely, the alcohol expectancy theory proposes that the self-fulfilling expectations of the effects of alcohol use may influence the user’s behavior [25]. For instance, those who believe alcohol use improves sexual performance and enhances sexual experiences may be more likely to consume alcohol while having sex. Furthermore, other studies have suggested that certain personality characteristics may predispose participation in risky sexual behaviors [26]. A study found that personality traits of sensation seeking and impulsive decision making are correlated with sexual risk taking [26, 27].

The purpose of this study was to estimate the prevalence of probable problematic alcohol use in comparison to no/low-risk alcohol use and identify associated factors in the Armed Forces of the Democratic Republic of the Congo (FARDC). Other objectives include examining associations between probable problematic alcohol use with the following risky sexual behaviors: sex with a sex worker, having multiple sexual partners, and participation in transactional sex. This study aimed to contribute to the paucity of research on alcohol use and risky sexual behaviors among military populations in Sub-Saharan Africa.

## Methods

### Study description and sampling

From October 2013 to April 2014, a seroprevalence and behavioral epidemiology risk survey (SABERS) was conducted in the FARDC [28]. The purpose of the SABERS was to determine the prevalence of HIV and syphilis and identify associated demographics and risk behaviors. Active duty men and women who were 18 years of age or older were eligible to participate in the cross-sectional study. To obtain a representative sample, 30 military bases from nine military regions were selected for study participation based on logistical feasibility. From these bases, military units were randomly selected for inclusion. The target sample size of the SABERS was proportional to the overall military size by rank. Convenience sampling was used to increase representation of senior officers (i.e., Captain, Major, and Colonel) as these individuals were typically affected by other competing military interests (i.e., prior commitments) that made random sampling difficult. Therefore, all available senior officers from the selected military units were invited to participate in the informational briefing. All other lower ranked service members were asked to draw a piece of paper from a hat – those who selected a marked piece of paper were invited to participate in the informational briefing. At the briefing, the study purpose, procedures, risks, and benefits were explained by a study team member. To ensure participants understood all aspects of the study, the consent form was available in either French or Lingala (a local dialect). All participants had the right to accept or decline participation in the SABERS and could stop the survey at any time without any negative consequences. All participants were given an opportunity to ask questions prior to giving electronic informed consent on a computer tablet. Compensation was not provided for participation in the study. Those who consented to take part in this study completed a computer-assisted personal interview performed by a trained study counselor in a private setting, and were then tested for HIV and syphilis.

The original research was conducted in compliance with all federal and local regulations and was approved by institutional review boards (IRBs) in San Diego, CA, USA (Naval Health Research Center [NHRC]) and Kinshasa, Democratic Republic of the Congo (Ministère de l’Enseignement Supérieur, Universitaire et Recherche Scientifique, Université de Kinshasa). Approval for secondary data analysis was obtained by the NHRC IRB.

### Variables and measures

The questionnaire was developed by epidemiologists at the United States Department of Defense HIV/AIDS Prevention Program and was adapted from similar studies conducted in other African militaries. Input from FARDC leadership was solicited and incorporated into the final survey, as well as feedback from FARDC service members who pretested the questionnaire for comprehension and cultural sensitivities prior to study implementation. The questionnaire was designed to collect information on demographics and HIV-related risk behaviors.

Demographic variables of interest included age, marital status, and education level. Marital status was categorized as single/widowed/divorced, single and living with a partner, or monogamous/polygamous marriage. Participants were asked to report the highest education level they completed. Responses were grouped as no school/completed less than primary, completed primary, completed secondary, or completed high school or university. Military characteristics of interest included rank, branch, and local deployments of more than six months in the past two years. Rank was categorized as recruit/soldier/corporal, non-commissioned officer, or officer. Military branch included the Army, Marine, Airforce, or other.

Hazardous, harmful, and dependent alcohol use was measured with the Alcohol Use Disorders Identification Test (AUDIT) [29]. The AUDIT was developed by researchers at the World Health Organization (WHO) and validated across different populations in six countries. It has been shown to accurately assess patterns of problematic alcohol use across age, gender, and cultures. WHO researchers defined hazardous alcohol use as a pattern of alcohol consumption that increased the risk of harmful consequences (e.g., physical, mental, or social) for the user or others. Harmful alcohol use was defined as a pattern of alcohol consumption that caused physical or mental damage to the user’s health and alcohol dependence referred to a cluster of behavioral, cognitive, and physiological symptoms that may develop after repeated use. These include a strong desire to consume alcohol, impaired control over its use, and a higher priority given to alcohol consumption compared to other activities [29]. The AUDIT consisted of 10 questions asking about recent alcohol use, alcohol dependence symptoms, and alcohol-related problems. A total score was computed from the questions which ranged from 0 to 40 and reflected a person’s level of risk related to alcohol. A score of 0 was an indicator of no alcohol use, 1 to 7 an indicator of low-risk alcohol use, 8 to 19 an indicator of probable hazardous and harmful alcohol use, and 20 or higher an indicator of possible alcohol dependence. In this study, WHO’s recommended cut-point of 8 for the AUDIT scale was used to identify individuals exhibiting potential alcohol problems: participants with scores of 8 or higher (defined as probable problematic alcohol use) were compared to those with scores of 7 or lower (defined as no/low-risk alcohol use).

Participants provided information on having sex with a sex worker, having multiple sexual partners, and engaging in transactional sex. Sex with a sex worker in the past 12 months was defined as sex with any person who provided sex in exchange for money. Multiple sexual partners was defined as participants who reported ever having sex with more than one sexual partner in the same week. Transactional sex was defined as ever paying or receiving money, shelter, food, drugs, favors or gifts in exchange for sex with a partner who was not a sex worker.

Participants also reported the influence of alcohol use on unintended sex and condom use in the past three months with the following questions: “In the last three months, did drinking alcohol influence your decision about or prevent you from using condoms or using condoms correctly? and “In the last three months, did you ever have unintended sex as a result of drinking alcohol?”

### Statistical analysis

Frequencies and percentages for categorical variables and means and standard deviations for continuous variables were computed. Bivariate logistic regression models were used to examine associations of demographic and military characteristics (i.e., categorical age, marital status, education level, rank, branch, and local deployment) with alcohol use. Several multivariable logistic regression models were used to independently examine the associations of alcohol use with sex with a sex worker, multiple sexual partners, and transactional sex after adjusting for categorical age, marital status, and education level. These variables were included as potential confounders based on previous literature [30, 31]. The multivariable models were restricted to those who reported ever having sexual intercourse which was defined as vaginal and/or anal sex, but did not include oral sex. Collinearity was assessed by evaluating tolerance values and the goodness-of-fit of the models was examined through Hosmer-Lemoshow tests. All tests were two-tailed, with *p* < 0.05 indicating statistical significance. Data analysis was conducted using SAS software, version 9.4 (SAS Institute Inc., Cary, NC, USA).

## Results

Of 2840 service members who were invited to the informational briefing, 2816 (99.2%) met eligibility criteria and consented to participate. Of these participants, 2751 (97.7%) completed the survey and biological testing. Due to the small number of women (*n* = 140), analyses were restricted to 2611 active duty male service members. Male service members who did not report ever having sexual intercourse (*n* = 61) or were missing data on participation in transactional sex (*n* = 1) were further excluded, resulting in a final sample size of 2549 men for the current study.

The mean age of these participants was 41.5 years (standard deviation = 10.3; range = 19 to 83) and the majority (40.0%) were in the 30 to 39 age group. Most men were married (either monogamous or polygamous) and had completed at least primary school (78.1 and 50.7%, respectively). With regards to military characteristics, the majority were ranked as a non-commissioned officer (41.4%) or officer (43.9%), and were serving in the Army (66.5%). Almost a quarter (20.2%) of respondents reported they had been on a local deployment lasting at least six months within the past two years. Fifteen percent (14.9%) of men screened positive for probable problematic alcohol use (AUDIT score ≥ 8) (Table 1).

**Table 1:** Characteristics of male FARDC service members (*N* = 2549)

Characteristic	Total (*N* = 2549)	
Age in years, mean (standard deviation)	41.5	(10.3)
Age group, *n* (%)		
19–29	239	(9.4)
30–39	1019	(40.0)
40–49	759	(29.8)
50+	532	(20.8)
Marital status, *n* (%)		
Single/widowed/divorced	203	(8.0)
Single and living with a partner	353	(13.9)
Monogamous/polygamous marriage	1993	(78.1)
Educational attainment, *n* (%)		
No school/completed less than primary	358	(14.0)
Completed primary	1291	(50.7)
Completed secondary	663	(26.0)
Completed high school or university	237	(9.3)
Military rank, *n* (%)		
Recruit/soldier/corporal	376	(14.7)
Non-commissioned officer	1055	(41.4)
Officer	1118	(43.9)
Military branch, *n* (%)		
Army	1696	(66.5)
Marine	122	(4.8)
Airforce	54	(2.1)
Other	677	(26.6)
Local deployment, *n* (%)		
Yes	516	(20.2)
No	2033	(79.8)
AUDIT score		
0 to 7: no/low-risk alcohol use	2169	(85.1)
8 or higher: probable problematic alcohol use	380	(14.9)

AUDIT: Alcohol Use Disorders Identification Test

Several variables were significantly associated with probable problematic alcohol use (Table 2). The odds of probable problematic alcohol use were elevated for men in the 30–39 and 40–49 age groups compared to men 50 or older (odds ratio [OR] 30–39 age group = 2.17; 95% confidence interval [CI] = 1.56–3.02; OR 40–49 age group = 1.79; 95% CI = 1.26–2.55). The odds of probable problematic alcohol use were significantly higher among participants who were single and living with a partner compared to those who were in a monogamous or polygamous marriage (OR = 1.66; 95% CI = 1.24–2.21). The odds were also elevated for those who were ranked as a non-commissioned officer compared to an officer (OR = 1.40; 95% CI = 1.10–1.77). No statistically significant associations were observed for education level, branch or local deployment (all *p* > 0.05).

**Table 2:** Factors associated with probable problematic alcohol use among male FARDC service members (*N* = 2549)

	Probable problematic alcohol use (*n* = 380)	No/low-risk alcohol use (*n* = 2169)		
Characteristic	n (%)	*n* (%)	OR (95% CI)	*P* value
Age group				< 0.0001
50+	50 (13.2)	482 (22.2)	ref (1.0)	
40–49	119 (31.3)	640 (29.5)	1.79 (1.26–2.55)	
30–39	187 (49.2)	832 (38.4)	2.17 (1.56–3.02)	
19–29	24 (6.3)	215 (9.9)	1.08 (0.65–1.80)	
Marital status				0.0015
Monogamous/polygamous marriage	271 (71.3)	1722 (79.4)	ref (1.0)	
Single and living with a partner	73 (19.2)	280 (12.9)	1.66 (1.24–2.21)	
Single/widowed/divorced	36 (9.5)	167 (7.7)	1.37 (0.94–2.01)	
Educational attainment				0.0804
Completed high school or university	27 (7.1)	210 (9.7)	ref (1.0)	
Completed secondary	93 (24.5)	570 (26.3)	1.27 (0.80–2.00)	
Completed primary	193 (50.8)	1098 (50.6)	1.37 (0.89–2.10)	
No school/completed less than primary	67 (17.6)	291 (13.4)	1.79 (1.11–2.90)	
Military rank				0.0180
Officer	145 (38.3)	973 (44.9)	ref (1.0)	
Non-commissioned officer	182 (47.8)	873 (40.2)	1.40 (1.10–1.77)	
Recruit/soldier/corporal	53 (13.9)	323 (14.9)	1.10 (0.79–1.55)	
Military branch				0.1709
Army	240 (63.2)	1456 (67.1)	ref (1.0)	
Marine	14 (3.7)	108 (5.0)	0.79 (0.44–1.40)	
Airforce	9 (2.4)	45 (2.1)	1.21 (0.59–2.51)	
Other	117 (30.7)	560 (25.8)	1.27 (0.99–1.61)	
Local deployment				0.4006
No	297 (78.2)	1736 (80.0)	ref (1.0)	
Yes	83 (21.8)	433 (20.0)	1.12 (0.86–1.46)	

*SD*: Standard deviation; *OR*: Odds ratio; *CI*: Confidence interval

With regards to risky sexual behaviors, 12.2% of all participants reported sex with a sex worker, 33.8% indicated having multiple sexual partners, and 35.2% participated in transactional sex. All of these behaviors were more commonly reported among participants with probable problematic alcohol use compared to those with no/low-risk alcohol use (Table 3).

**Table 3:** Risky sexual behaviors by alcohol use among male FARDC service members (*N* = 2549)

	Total (*N* = 2549)	Probable problematic alcohol use (*n* = 380)	No/low-risk alcohol use (*n* = 2169)
Risky Sexual Behavior	n (%)	n (%)	n (%)
Sex with a sex worker			
No	2237 (87.8)	292 (76.8)	1945 (89.7)
Yes	312 (12.2)	88 (23.2)	224 (10.3)
Multiple sexual partners			
No	1687 (66.2)	192 (50.5)	1495 (68.9)
Yes	862 (33.8)	188 (49.5)	674 (31.1)
Participated in transactional sex*			
No	1652 (64.8)	189 (49.7)	1463 (67.5)
Yes	897 (35.2)	191 (50.3)	706 (32.5)

* Paid or received money, shelter, food, drugs, favors, or gifts in exchange for sex

Probable problematic alcohol use was found to be significantly associated with sex with a sex worker, multiple sexual partners, and transactional sex (Table 4). After adjusting for age, marital status, and education level, the odds of having sex with a sex worker were two times higher among men with probable problematic alcohol use compared to men with no/low-risk alcohol use (adjusted odds ratio [aOR] = 2.36; 95% CI = 1.78–3.13). The odds of having multiple sexual partners and participating in transactional sex were also elevated among men with probable problematic alcohol use compared to men with no/low-risk alcohol use (aOR multiple sexual partners = 2.08; 95% CI = 1.66–2.60; aOR transactional sex = 1.99; 95% CI = 1.59–2.50). Results from the Hosmer-Lemeshow tests indicated a good fit for each model. Additionally, multicollinearity was not an issue.

**Table 4:** Adjusted associations of probable problematic alcohol use with risky sexual behaviors among male FARDC service members (*N* = 2549)

Risky Sexual Behaviors	AOR* (95% CI)	*P* value
Had sex with a sex worker		
Yes vs. No	2.36 (1.78–3.13)	< 0.0001
Had multiple sexual partners		
Yes vs. No	2.08 (1.66–2.60)	< 0.0001
Participated in transactional sex**		
Yes vs. No	1.99 (1.59–2.50)	< 0.0001

AOR: adjusted odds ratio.

*Adjusted for age, marital status, and education level

** Paid or received money, shelter, food, drugs, favors, or gifts in exchange for sex

Of participants who had sex and drank alcohol in the past three months (*n* = 1209), 14.3% (*n* = 173) reported alcohol consumption influenced their decision about using condoms or prevented them from using condoms correctly. A total of 18.4% (*n* = 223) also reported unintended sex as a result of drinking alcohol (results not shown in a table).

## Discussion

There is mounting evidence suggesting that alcohol use is an important factor to address in the HIV epidemic. Previous research has shown that patterns of problematic alcohol consumption are more likely to occur in regions that also have the highest burden of HIV disease, notably in eastern and southern Africa [32]. With projections that the alcohol market is anticipated to flourish substantially in this region due to growing economies and urbanization [33], identification of priority populations, such as militaries, who may be at higher risk for alcohol misuse is highly important. This study identified factors associated with probable problematic alcohol use in male FARDC service members and evaluated associations of misuse with risky sexual behaviors.

A considerable proportion (14.9%) of male service members in the FARDC screened positive for probable problematic alcohol use. This estimate is almost two times higher than rates reported among men in the general population of the Democratic Republic of the Congo (9.1%). These findings are comparable to other studies that have also found alcohol misuse to be substantially higher among military populations compared to their civilian counterparts [21, 22]. There are several possible explanations for higher rates of alcohol misuse among service members. Historically, alcohol use has been a part of military culture as a way to alleviate stress [34–36]. Service members in the FARDC may also have more disposable income than their civilian counterparts, which may be used to purchase alcohol. Additionally, there are unique circumstances of military service that may be associated with problematic drinking including exposure to combat and traumatic events [37–40] and frequent and lengthy deployments from loved ones [21]. Comprehensive measures must be taken to reduce alcohol misuse among male FARDC service members. Military institutions have the ability to implement policies that can change norms around alcohol use. For example, they can refuse to sell alcohol on base, or limit its sale to certain hours or days of the week. Military health systems can also include screening for problematic alcohol use during annual health screens to determine fitness for duty. Men who screen positive for probable problematic alcohol use should be referred to a military or civilian medical center for treatment. Furthermore, social marketing campaigns can take advantage of perceptions that military members are ‘tough’ to promote messages that unsafe alcohol use may interfere with physical fitness.

Several demographic and military factors were found to be correlated with probable problematic alcohol use including being single and living with a partner, which is consistent with previous studies in other militaries [21, 41–43]. Post-hoc analyses found that men who were single and living with a partner were substantially younger than men who were married (mean age of 35.8 years old vs. mean age of 43.1 years old). These participants may have less responsibilities (e.g., no children) and therefore may be more likely to have free time to engage in social activities that involve drinking. Higher rank (i.e., rank of non-commissioned officer) was also associated with probable problematic alcohol use. The FARDC should hold a workshop with higher leadership to address alcohol misuse and its detrimental effects on the individual and overall force health. The FARDC should also draft a policy on alcohol management and distribute this policy to higher leadership. Having senior leaders lead by example may have a positive outcome on service members they are responsible for overseeing. Age was also associated with alcohol misuse. The odds of probable problematic alcohol use were highest among men who were in the 30 to 49 age groups. While previous studies have shown alcohol misuse to be a problem among service members 17 to 25 years of age [41] and those 20 to 29 [42, 43], this study demonstrated that probable problematic alcohol use could still be an issue among older age groups. In summary, these findings suggest that certain characteristics place military personnel at increased risk for probable problematic alcohol use. Reducing and preventing dangerous alcohol consumption must be a priority for FARDC leadership to maintain operational readiness. Comprehensive alcohol misuse education would benefit all forces, but efforts should be targeted at men who are 30 to 49 years of age, not married, and of higher rank.

Probable problematic alcohol use was found to be associated with having sex with a sex worker, engaging in multiple sexual partners, and participating in transactional sex among male service members in the FARDC. These findings are consistent with previous literature conducted in other military populations [21, 44]. Furthermore, a substantial percentage of participants reported that alcohol had a direct effect on their sexual behaviors: 14.3% reported alcohol consumption influenced their decision about using condoms or prevented them from using condoms correctly and 18.4% reported alcohol use led to unintended sex. While the FARDC does have a comprehensive HIV prevention program, these data highlight the need to incorporate alcohol abuse prevention into the existing program and underscore its role in HIV transmission and acquisition through risky sexual behaviors. Additionally, other preventive measures include the promotion of social events that don’t involve alcohol consumption (e.g., participation in sports).

Several study limitations are noted. All data were self-reported and subject to recall and social desirability bias. However, interviewers were trained in proper data collection techniques, and all respondents were instructed they could stop the survey at any time or not answer any questions they did not feel comfortable providing a response. Comparisons of probable problematic alcohol use in the military and civilian counterparts should be interpreted with caution as different measurements for alcohol misuse may have been used: the AUDIT was used in this study, but it is unclear what measurement was used in the WHO global report on alcohol use [45]. Due to the cross-sectional nature of the study, temporality and causality between variables cannot be determined. While strong associations between probable problematic alcohol use and risky sexual behaviors were observed, temporality and the causal relationship remains inconclusive for various reasons. Residual confounding is plausible as other factors (e.g., personality traits and mental health problems) that may affect these associations were not measured in the SABERS. Reverse causality is plausible as well; participants may have used alcohol as a mechanism to cope with participation in risky sexual behaviors. Further studies are needed to elucidate the causal relation between alcohol use and risky sexual behaviors. Convenience sampling of senior officers may have introduced selection bias into the SABERS study and results should be interpreted with this limitation in mind. Due to the small number of women who participated in the original SABERS study, analyses among female service members were not performed and therefore study results are not generalizable to this group. Further studies among female service members are needed to characterize the relation between alcohol use and risky sexual behaviors.

## Conclusions

These data add to growing research among African military populations that alcohol use may fuel the HIV epidemic by increasing risky sexual behaviors. Identifying specific subsets of the FARDC population who are more likely to misuse alcohol is an important first step for FARDC leadership to implement a comprehensive alcohol misuse prevention program that is tailored to those at highest risk. Preventing alcohol abuse, screening for problematic use, and referring those who have exhibited patterns of excessive drinking to medical care and treatment are critical activities to incorporate into existing HIV prevention programs. Additionally, developing effective messages and interventions to mitigate harmful and hazardous alcohol use in relation to risky sexual behaviors are essential to maintain troop health and preserve force readiness.

## Data Availability

The datasets generated and/or analyzed during the current study are not publicly available due to the sensitive and confidential nature of the data as determined by the HIV/AIDS Directorate of the Armed Forces of the Democratic Republic of the Congo. Requests for data access should be made to Colonel Anthony Mbuyi (mbuyimutombeanthony@yahoo.com) and Bonnie Tran (bonnie.r.tran.ctr@mail.mil).

## Abbreviations

AUDIT: Alcohol Use Disorders Identification Test
DHAPP: Department of Defense HIV/AIDS Prevention Program
FARDC: Armed Forces of the Democratic Republic of the Congo
IRB: Institutional Review Board
NCO: Non-commissioned officer
NHRC: Naval Health Research Center
SABERS: Seroprevalence and Behavioral Epidemiology Risk Survey
SW: Sex worker
WHO: World Health Organization
